# Conventional, Hybrid, or Electric Vehicles: Which Technology for an Urban Distribution Centre?

**DOI:** 10.1155/2015/302867

**Published:** 2015-07-05

**Authors:** Philippe Lebeau, Cedric De Cauwer, Joeri Van Mierlo, Cathy Macharis, Wouter Verbeke, Thierry Coosemans

**Affiliations:** MOBI Research Group, Vrije Universiteit Brussel, Pleinlaan 2, 1050 Brussels, Belgium

## Abstract

Freight transport has an important impact on urban welfare. It is estimated to be responsible for 25% of CO_2_ emissions and up to 50% of particles matters generated by the transport sector in cities. Facing that problem, the European Commission set the objective of reaching free CO_2_ city logistics by 2030 in major urban areas. In order to achieve this goal, electric vehicles could be an important part of the solution. However, this technology still faces a number of barriers, in particular high purchase costs and limited driving range. This paper explores the possible integration of electric vehicles in urban logistics operations. In order to answer this research question, the authors have developed a fleet size and mix vehicle routing problem with time windows for electric vehicles. In particular, an energy consumption model is integrated in order to consider variable range of electric vehicles. Based on generated instances, the authors analyse different sets of vehicles in terms of vehicle class (quadricycles, small vans, large vans, and trucks) and vehicle technology (petrol, hybrid, diesel, and electric vehicles). Results show that a fleet with different technologies has the opportunity of reducing costs of the last mile.

## 1. Introduction

A number of trends can be observed in urban freight transport. The current urbanization process generates more freight volumes in cities, transport is increasingly fragmented due to the success of light commercial vehicles, and distances are stretching out due to the delocalisation of logistics platforms to the periphery [1]. Because of these combined effects, vehicle-kilometres of freight vehicles are expected to increase in the future. However, urban freight transport is responsible for negative impacts on the sustainability of cities. These negative impacts can be attributed partly to the intense use of road. Vans and trucks have indeed a worse impact compared to other motor vehicles such as cars and motorcycles [2]. Even though road freight transport represents 10 to 15% of vehicle-kilometres in cities [3, 4], freight vehicles are responsible for around 25% of CO_2_ emissions, 30% of NO_*x*_ emissions, 40% of energy consumption, and 50% of particles matter [5, 6]. Also, noise nuisance caused by freight transport generates around five times more decibels than the circulation noise of private cars during morning rush hour [4]. Recognizing the need for solutions, the European Commission has set the objective of reaching free CO_2_ city logistics in major urban areas by 2030 [7].

**Figure 1:**
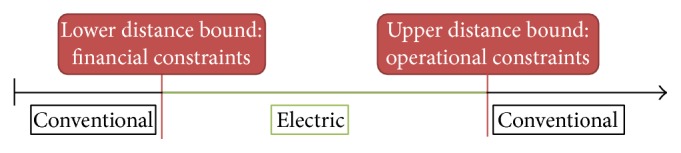
The paradox of the battery electric vehicle. Source: own setup.

Research has developed a wide range of logistics concepts, regulations, and technologies to fulfil the city logistics carbon-free target [8]. Among them, battery electric vehicles (BEVs) are considered to be an answer to the negative impacts listed above [9]. They have a particularly low environmental impact compared to conventional vehicles [10]. Several big companies such as DHL, UPS, DPD, and Japan Post have already integrated BEVs in their fleet for last mile deliveries [11]. Still, purchase costs and limited battery capacity remain the two most important barriers for BEV adoption [12]. They both contribute to the paradox of the BEV as depicted in Figure 1. On the one hand, they have to drive a high number of kilometres to be competitive with conventional vehicles. On the other hand, range is limited due to the battery capacity. As a result, BEVs fit in a specific niche. The objective of this paper is to address these constraints by comparing the use of battery electric vehicles with conventional vehicles in a delivery fleet.

Based on a vehicle routing problem (VRP) that we formulated, we study the case of an urban distribution centre. It will use the (real) case of a distributor which has a depot located in Brussels. The constraint related to the limited range defines the area of possible solutions. The optimal solution is then identified by the composition of the fleet that shows the lowest total cost.

## 2. Literature Review

The organisation of delivery tours has generally been investigated through the vehicle routing problem (VRP). It is defined as “the determination of the optimal set of routes to be performed by a fleet of vehicles to serve a given set of customers” [13]. The optimisation can have different objectives such as the minimisation of traveling time or delivery costs. The VRP can be described as a travelling salesman problem (TSP) where more than one vehicle is used to serve each customer. The original VRP was introduced by Dantzig and Ramser [14] and then developed in a large variety of more complex versions.

Constraints on limited driving range were introduced with the works of Christofides et al. [15]. The VRP was designed such that a maximum cost could not be exceeded by the solution. The maximum costs parameter could be either replaced by time constraints or by distance constraints. This idea was also used by Laporte et al. [16] where distance travelled by any vehicle could not exceed a defined upper bound. They named it the distance constrained vehicle routing problem (DCVRP) and kept improving it in later works such as in Laporte et al. [17, 18].

The first attempt to investigate the specific characteristics of BEVs in a VRP was achieved by Gonçalves et al. [19]. They considered a VRP with pickup and delivery (VRPPD) and a mix fleet made of BEVs and conventional vehicles. The limited battery capacity was represented by a time constraint on charging BEVs. The approach enriched the previous work on DCVRP as the distance constraints can be extended against a loss in time due to charging the battery. However, the locations of charging spots were not considered in the model, meaning that BEVs could virtually recharge anywhere on the delivery round once the battery was empty. Erdoğan and Miller-Hooks [20] brought a solution to the weaknesses of Gonçalves et al. [19] by developing the green VRP (G-VRP). They consider a network of refuelling stations that alternative fuelled vehicles can use during their delivery tour. They based their mixed integer linear program formulation on the VRP with satellite facilities (VRPSF) from Bard et al. [21]. They translated the concept of satellites facilities where the cargo of vehicles can be reloaded or unloaded during the route into charging spots where vehicles can be refuelled during the route at specific points in the network.

Erdoğan and Miller-Hooks [20] showed therefore how to consider the location of refuelling in a VRP, though the charging time considered by Gonçalves et al. [19] is missing in the G-VRP as it was not designed specifically for BEVs but for alternative fuel vehicles (i.e., biodiesel, liquid natural gas, or CNG vehicles). The contributions of Gonçalves et al. [19] and Erdoğan and Miller-Hooks [20] were integrated by Schneider et al. [11] in their electric vehicle routing problem with time windows (E-VRPTW). Charging locations and charging times are both considered in their model which approaches well the problem of BEVs. In particular, they modelled charging time of BEVs as being a function of the state of charge of the battery. Moreover, time windows and vehicle capacity restrictions are also included in the constraints of the E-VRPTW in order to adapt the model to the context of urban freight distribution.

At the same time, Conrad and Figliozzi [22] developed a solution close to that of Schneider et al. [11]. Based on a capacitated vehicle routing problem with time windows constraints (CRVRP-TW), they introduced the limited range and charging times in order to get the recharging vehicle routing problem (RVRP). Their main difference is regarding charging locations: Conrad and Figliozzi [22] consider that charging is possible at some customer locations while the formulation of Schneider et al. [11] is more flexible as other possible charging locations are possible in the network.

So far, these different papers considered VRP with a single type of vehicle. However, electric vehicles are likely to be used in delivery fleets with other kinds of vehicles. A well-studied branch of the VRP literature is precisely addressing the problem of heterogeneous fleets in delivery fleets [23]. Merging the VRP research on electric vehicles with the fleet size and mix vehicle routing problem (FSMVRPTW) is therefore relevant to come with recommendations for logistics decision makers. van Duin et al. [24] have been the first to develop this idea with their electrical vehicle fleet size and mix vehicle routing problem with time windows (EVFSMVRPTW). However, they approached the problem from the FSMVRPTW branch without considering the previous work on battery electric vehicles in VRP. As a result, the model was entailed with similar weaknesses than in Gonçalves et al. [19]: they do not consider the locations of charging points. A BEV with a battery swapping system is modelled so that the range of this BEV can be doubled. But the swapping system is not reflected in the constraints. It is in fact reflected in the range parameter of the vehicle which is simply doubled, meaning that the battery of the BEV can be swapped virtually anywhere on the road. Still, the main benefit of van Duin et al.'s work [24] is bringing the fleet size and mix approach in the discussion of electric vehicle routing problem. Hiermann et al. [25] developed that idea further to propose an E-FSMVRPTW that considers the decisions regarding the fleet composition and the choice of recharging times and locations. This work can be considered as the state of the art of delivery optimisation with BEVs.

Table 1 summarises the contributions of the different relevant papers. However, one aspect is forgotten in every paper. All BEV specific papers assume the range to decrease linearly in function of the distance driven. However, the literature from engineering research recognises that range of BEVs is strongly influenced by other parameters than distance. Hayes et al. [26] show, for example, in their paper that the driving range for a specific BEV (Nissan Leaf) can change from 221 km in ideal driving conditions to 99 km in bad conditions. In order to facilitate for consumers the comparison between BEVs' performances, manufacturers have to show the range based on official drive cycles. In the United States, the EPA is used and shows a range of 121 km for the Nissan Leaf [27]. In Europe, the NEDC is used and shows a range of 200 km for the Nissan Leaf [28]. Hence, range can change to a large extent depending on the usage of the vehicle. Two current works are integrating vehicle dynamics in a VRP to estimate variable ranges [29, 30]. However, more technical knowledge is required to develop an energy model. Auxiliaries, for example, were not considered even though they represent an important part of the energy consumption.

Since Lin et al. [23] identify precisely the lack of interdisciplinary approach for solving VRP problems, the objective of this paper is to bring together the developments of the EVFSMVRPTW with real observations conducted on electric vehicles. The model we propose considers therefore the different aspects shown in Table 1. The fleet size and mix vehicle routing problem considers different vehicle sizes with either electric propulsion or internal combustion engine. They mainly differ by their payload, fixed costs, running costs, energy available in the vehicle, and their energy consumption. Charging operations are considered for battery electric vehicle at the depot with fast chargers. Finally, time windows are also considered as they are important to be considered in the context of city distribution. As a result, we call our formulation of the problem a fleet size and mix vehicle routing problem with time windows for electric vehicles (FSMVRPTW-EV).

## 3. Methodology

### 3.1. The Parameters Influencing the Range of BEV

The range of an electric vehicle is determined by the amount of energy at disposal in the battery and the energy consumption of the vehicle. The available total energy in the batteries for vehicles is called the battery capacity and the remaining amount of energy during use is called the state of charge (SoC) and is expressed in percentage “charge” remaining.

The energy required at the wheels to drive a vehicle is determined by the vehicle dynamics. Based on El Baghdadi et al. [31], we can express a theoretic energy consumption *E*_*ij*_ at the wheels for a distance *d*_*ij*_ using the vehicle dynamics described in

(1) Eij=13600dbdtmij·g·ω·cosφ+sinφ  13600+0.0386·ρ·σ·μ·bij2  13600+mij+mf·dbdtdij,

where *E*_*ij*_ is mechanical energy required at the wheels to drive on a distance *d*_*ij*_ (kWh), *m*_*ij*_ is vehicle mass (kg), *m*_*f*_ is fictive mass of rolling inertia (kg), *g* is gravitational acceleration (9.81 m/s²), *ω* is vehicle coefficient of rolling resistance (-), *φ* is road gradient angle (°), *ρ* is air density (1.226 kg/m^3^), *σ* is drag coefficient of the vehicle (-), *μ* is max. vehicle cross-section (m²), *b*_*ij*_ is vehicle speed between the point *i* and the point *j* (km/h), and *d*_*ij*_ is distance driven from point *i* to point *j* (km).

The first term of the formula assesses the rolling resistance due to the work of deformation on wheel from the contact with the road. It also considers the required potential energy for hill climbing. The second term assesses the aerodynamic drag (losses), which are heavily dependent on the shape of the vehicle and the driving speed. Finally, the third term considers the energy required for acceleration. By combining these three factors, we can estimate the theoretical force to move the vehicle. If we consider the distance on which this force is applied, we compute the energy required to move the vehicle.

**Figure 2:**
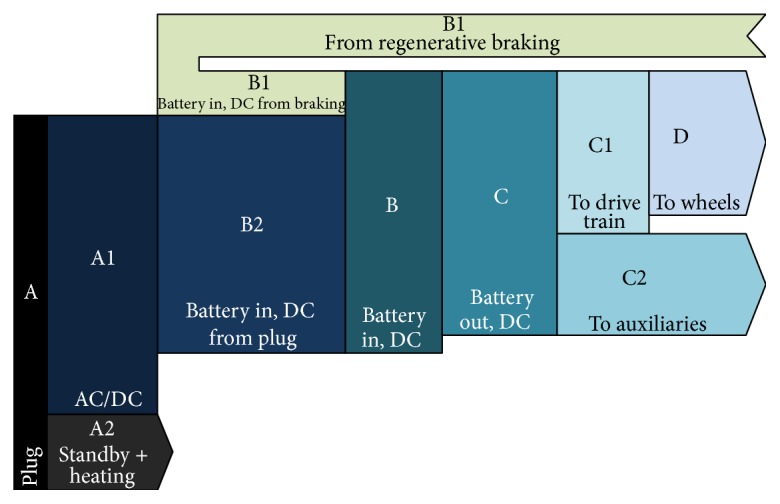
Energy losses from plug to the wheels of battery electric vehicles. Source: [32].

Driving the vehicle is however not the only source of energy consumption. Auxiliaries (AC, heating, etc.) represent another important part of energy consumption. Additionally, to deliver energy from the battery to the wheels and auxiliaries, the energy is submitted to a number of conversion stages, each comprising energy losses, as estimated by De Vroey et al. [32]. On the other hand, the amount of available energy is increased by the ability of the electric vehicle to recover (regenerated) part of the kinetic (braking) or potential (hill descend) energy.  Figure 2 shows how energy is transmitted from the grid to the wheels and how it is distributed between the drive train and auxiliaries in the final stage.

An accurate range model is therefore the combination of an accurate SoC estimation and an accurate energy consumption estimation. As energy consumption varies considerably with changing circumstances, a large impact on the vehicle's range is expected. Therefore, a first energy consumption model using real-life BEV consumption measurements and the vehicle dynamics can result in a more realistic range estimation of BEVs in the VRP.

### 3.2. Data Collection and Assumptions

In order to model the energy consumption of BEVs in the FSMVRPTW-EV, we used real observations of the energy consumption of a Nissan Leaf collected from December 2012 until December 2013. For each trip, duration, distance, and date were monitored. Most importantly, the energy consumed and recovered during the trip was registered. Since the car was shared between different drivers, a variety of driving behaviours could also be observed in our data. After filtering the sample, eliminating very short trips (less than 1 km) and corrupted data, we kept 838 observations for analysis.

Based on these data, we modelled the energy consumed through an ordinary least squares analysis. We considered the theoretical relationships described in the previous section to explain the energy consumption. Hence, before the ordinary least square analysis is conducted, the theoretical energy required by the electric drivetrain was estimated for each trip based on vehicle dynamics. But some information was missing in the description of the trips such as acceleration and road gradient. Also, energy losses when converting electrical energy to mechanical is missing. This information should be however considered in the model through the error term or the coefficient *β* of the ordinary least square analysis. We also assumed auxiliary consumption to be a function of time. Finally, since temperature affects the consumption of both auxiliary and drivetrain efficiencies, an additional parameter is included for correctional purposes. As a result, we explain the energy of a trip according to the function described in

(2) EnergyConsumptiontrip=α∗Durationtrip+β∗Eij +γ  Temperaturetrip+ε.

**Figure 3:**
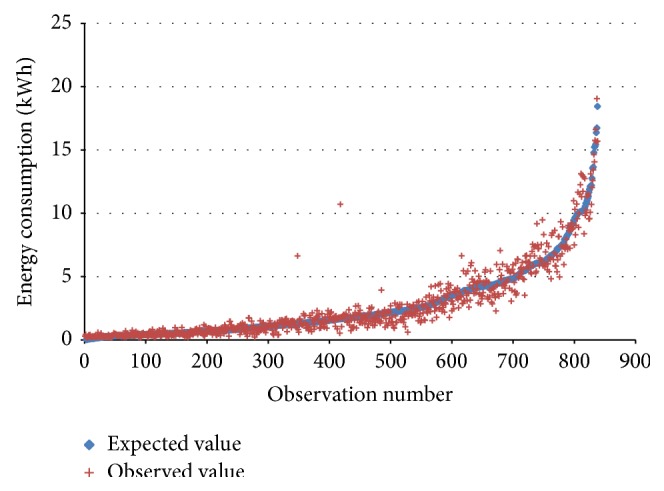
Observed and expected energy consumption of electric vehicle trips. Source: own setup.

The nonlinear least square analysis showed that the variables chosen were very significant with a *P* value of 2.39*e* − 113, 9.42*e* − 235, 5.44*e* − 6, and 7.42*e* − 5 for, respectively, *α*, *β*, *γ*, and *ε*. The model is also assessed to be excellent with a *R* ^2^ of 0.93. In Figure 3, we can see the distribution of the expected and the observed values.

As the section above described, there is also a positive flow of energy with regenerative braking. In order to model the contribution of the regenerated energy, we developed also a model through an ordinary least squares analysis. We used the function described in (3) to explain the regenerative energy in function of duration of the trip, distance, and the temperature:

(3) RegenaritveEnergytrip  =δ∗Distancetrip+θ∗Temperaturetrip   +π∗Durationtrip+τ.

**Figure 4:**
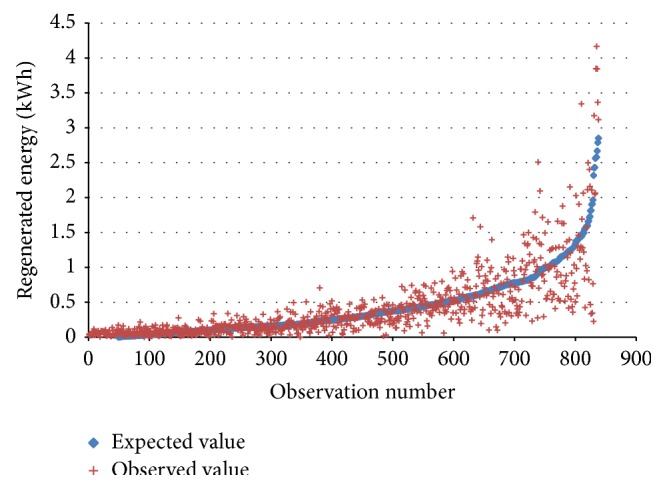
Observed and expected regenerated energy of electric vehicle trips. Source: own setup.

Each variable was assessed to be very significant with a respective *P* value of 2.73*e* − 33, 3.59*e* − 8, and 3.97*e* − 45. The constant error term had also a very low *P* value with 2*e* − 8.  Figure 4 shows the distribution of observed and expected values of the regenerated energy. The *R* ^2^ is 0.77.

By combining both models in the FSMVRPTW-EV, we can estimate the depletion of the battery in function of the route taken by the BEV. The energy capacity of the battery vehicle is reduced by its specific consumption on each route. Let us note that this specific consumption considers the average driving behaviour from our observations.

### 3.3. The FSMVRPTW-EV

The formulation of the fleet size and mix vehicle routing problem with time windows for electric vehicles (FSMVRPTW-EV) is based on the FSMVRPTW of Belfiore and Yoshizaki [33] and the G-VRP of Erdoğan and Miller-Hooks [20]. It is defined on a complete and directed graph *G* = (*V*, *A*). *V* denotes a set of vertices with *V* = *C* ∪ {*v*_0_}. *C* is a set of *n* customers with *C* = {*v*_1_, *v*_2_,…, *v*_*n*_} and {*v*_0_} stands for the depot. Then, set *A* represents the set of arcs connecting the vertices of *V*, with *A* = {(*v*_*i*_, *v*_*j*_)∣*v*_*i*_, *v*_*j*_ ∈ *V*, *i* ≠ *j*}. Each arc (*v*_*i*_, *v*_*j*_) is associated with a distance *d*_*ij*_, a speed *b*_*ij*_, and a traveling time *t*_*ij*_.

*P* represents a set of *k* vehicles with *P* = {*p*_1_, *p*_2_,…, *p*_*k*_}. They are either BEVs or conventional vehicles. Hence, they have different properties. They differ according to their fixed costs *f*_*k*_, their running costs *g*_*k*_, their payload *m*_*k*_, and their volume capacity *a*_*k*_. They differ also according to their energy capacity *z*_*k*_ and the energy consumption *h*_*ij*_^*k*^ they spend to travel from *v*_*i*_ to *v*_*j*_. The energy capacity is reduced by 10% of the announced battery capacity to take into account the maximum depth of discharge. The energy consumption is based on the range model presented in the previous section. On the other hand, the vehicles share common characteristics. The driver cost *c* (*€*/hour) remains the same across vehicles. Vehicles start and end at the depot *v*_0_. They travel in the directed graph *G* so that the demand of every customer is fulfilled. Demand is described both in terms of volume with *q*_*n*_ and in terms of weight with *o*_*n*_. Each vertex of *C* is associated with a time window [*l*_*i*_, *u*_*i*_] and a service time *s*_*i*_. Deliveries cannot start before *l*_*i*_ and after *u*_*i*_ but can end after *u*_*i*_ given the service time *s*_*i*_. Once the vehicle *k* has come back to the depot, the used vehicle can do additional routes and become vehicle *k*′. Hence, *P*′ denotes the set of used vehicles. Vehicles in *P*′ have a fixed cost of zero since it has already been considered in their first route, but they are available later. Let us note that recharging is possible for BEV at the depot only. BEV can fast charge with a power *r* during their loading/unloading operations that we consider set at 50 kW. In order to optimise the lifetime performance of the batteries, they can reach a maximum state of charge of 80% of their initial capacity. We assume in our model that a fast charger is always available at the depot.

The objective of the FSMVRPTW-EV is to minimise the total costs of fulfilling the demand of customers within their time windows. The binary decision variables *x*_*ij*_^*k*^∣*k* ∈ *P*,  *i*, *j* ∈ *V*,  *i* ≠ *j* represent the resulting route followed by the vehicles such that *x*_*ij*_^*k*^ equals 1 if the arc (*i*, *j*) has travelled and 0 otherwise. Besides, vertices are associated with additional decision variables: *e*_*i*_^*k*^ shows the available energy of vehicle *k* at customer *i*,  *a*_*i*_^*k*^ gives the available volume capacity of vehicle *k* at customer *i*,  *m*_*i*_^*k*^ denotes the available payload of vehicle *k* at customer *i*, and *w*_*i*_^*k*^ gives the time of arrival of vehicle *k* at customer *i*.

*Indices and Sets*

i,j: Vertex indices
V: Set of all vertices with *V* = *I* ∪ {*v*_0_}
C: Set of *n* customers with *C* = {*v*_1_, *v*_2_,…, *v*_*n*_}
A: Set of arcs with *A* = {(*v*_*i*_, *v*_*j*_)∣*v*_*i*_, *v*_*j*_ ∈ *V*,  *i* ≠ *j*}
P: Set of *k* vehicles with *P* = {*p*_1_, *p*_2_,…, *p*_*k*_}.

*Parameters*

$v_{n}$: The customer *n* with *v*_*n*_ ∈ *I*
$v_{0}$: Depot with *v*_0_ ∈ *V*
$q_{n}$: Volume of goods to be delivered at customer *n* (m³)
$o_{n}$: Weight of goods to be delivered at customer *n* (kg)
$l_{n}$: Lower bound of the time windows for customer *n* (h-time)
$u_{n}$: Upper bound of the time windows for customer *n* (h-time)
$s_{n}$: Service time to deliver customer *n* or to load the vehicle if *n* = 0 (h-duration)
$d_{ij}$: Distance from node *i* to node *j* (km)
$b_{ij}$: Speed limit between node *i* and node *j* (km/h)
$t_{ij}$: Time of travel between node *i* and node *j* (h-duration)
$p_{k}$: The vehicle *k* with *p*_*k*_ ∈ *P*
$a_{k}$: Volume capacity of the vehicle *k* (m³)
$m_{k}$: Payload of the vehicle *k* (kg)
$e_{k}$: Maximum available energy capacity of the vehicle (kWh)
$g_{k}$: Running costs of vehicle *k* (*€*/km)
$f_{k}$: Fixed cost of vehicle *k* (€)
c: Cost for the driver (*€*/h)
r: Charging power at the depot (kW).

*Variables*

$w_{i}^{k}$: Arrival time of vehicle *k* at node *i* (h-time)
$e_{i}^{k}$: State of charge of vehicle *k* at node *i* (kWh)
$h_{ij}^{k}$: Energy consumed by vehicle *k* from node *i* to node *j* (kWh)
$a_{i}^{k}$: Goods' volume being transported by vehicle *k* at node *i* (m³)
$m_{i}^{k}$: Goods' weight being transported by vehicle *k* at node *i* (kg)
$x_{ij}$: Binary variable to 1 if the arc (*i*, *j*) is travelled, 0 otherwise.

The formulation of the FSMVRPTW-EV can be expressed as the following mixed-integer program:

(4) Minimize ∑k∈P ∑j∈Cfkx0jk+∑i∈V,i≠j ∑j∈V,i≠j ∑k∈Pdijkxijkgk

(5) Subject  to ∑j∈Cx0jk=1, ∀k∈P,       

(6)       ∑i∈V,i≠jxijk−∑i∈V,i≠jxjik=0, ∀j∈V,  ∀k∈P,

(7)       ak≥ajk−qj+Mij1−xijk≥aik≥0,

(8)       mk≥mjk−oj+Mij1−xijk≥mik≥0,

(9)       ek≥eik−hijk+Mij1−xijk≥ejk≥0,

(10)     wik+si+tij−Mij1−xijk≤wjk,

(11)       li≤wik≤ui, ∀i∈V,  ∀k∈P,

(12)       e0k≤1−0.10∗ek, ∀k∈P,  ∀i∈V,

(13)       e0k≤s0∗r≤0.80−0.10∗ek,

(14)       xijk∈0,1, ∀k∈P,  ∀i,j∈V,  i≠j.

Equation (4) represents the objective function. It expresses the total costs associated with a solution of the FSMVRPTW-EV. The first term considers the fixed costs of vehicles leaving the depot. The second term computes the running costs of the vehicles. Finally the last term considers the staff costs to operate the vehicle.

Constraints are given by (5) to (14). Constraints (5) and (6) guarantee that vehicles start from the depot, visit the customers, and come back to the depot. Constraint (6) in particular ensures the conservation of flow by forcing the equality between the number of arrivals and the number of departures at each vertex. Constraints (7) and (8) guarantee that the vehicle capacity is not exceeded in terms of volume and weight. They also track their reduction through the deliveries. *M*_*ij*_ is a sufficiently large number. Constraint (9) guarantees that the available energy is always positive and tracks the battery depletion through the route. Constraint (10) sets a minimum arrival time for vehicle *k* arriving at customer *j*. Constraint (11) ensures that customers are visited during their time windows and that vehicles are operated during the opening hours of the depot. Constraints (12) and (13) consider the maximum depletion of the battery. In particular, constraint (13) considers fast charging possibilities at the depot. Finally, constraint (14) ensures the binary integrality.

### 3.4. The Algorithm

The algorithm we developed to solve the FSMVRPTW-EV is based on the savings heuristic [34]. First, an initial solution is built with each shop being delivered by one route. Subsequently the solution is iteratively improved by searching for potential savings by merging two or more routes.

In order to address the constraints of the FSMVRPTW-EV some adjustments have been made to the original savings heuristic algorithm. When searching for potential savings, the adjusted algorithm limits the computation of potential savings to the five closest shops to the last shop inserted in the route. When comparing with an algorithm searching for savings for all shops, results are barely changed whereas computation times are reduced drastically. Moreover, as proposed by Bräysy et al. [35], the idea of the insertion-based heuristic is used. When merging an initial route (thus delivering to only one shop) with the route being improved, each possible position of the shop in the route is considered. The position showing the largest savings is selected and another initial route is investigated for merging. As a result, the shop to be inserted is the one showing the largest savings when located at the best position in that route.

Bräysy et al. [35] also recommend using an insertion sequence for customers. The algorithm is more effective when the most critical shops are investigated first (i.e., shops with short time windows). The algorithm starts to build routes around the most difficult shops to insert. If these would be inserted later, once most shops are already included in the constructed routes, there might be no possible insertion left for that shop. The algorithm would then create a single route delivering only to that critical shop which is not cost effective. The insertion sequence is therefore based on a criticality index that considers for each shop the start of the time window, the time window duration, and the distance from the depot. Shops that are inserted first are shops with a limited time window, starting early and located closer to the depot.

In order to consider the different combinations of vehicles, the algorithm uses a tree. In each branch, one of the vehicles is selected. According to the heuristic described above, a maximum of shops are inserted in the route, given the selected vehicle. The resulting node of that branch shows the number of shops that still need to be delivered. The branch is then developed further until all the shops are delivered. We have then a possible solution. Still, if the total cost of a branch becomes higher than the total cost of a completed branch, then the branch is not further explored. Finally, once the tree is fully built, the branch with the lowest cost is considered as the optimal solution.

### 3.5. Instances

Benchmark instances are common practice to compare performance of different algorithms. However, they use mainly Euclidian distances to assess distances between shops. Since speed becomes a factor influencing the range of BEVs, O-D matrices between shops in terms of distance and time are required to feed the energy model.

We generated a set of instances based on a real case in urban freight transport. Distribution occurs from an urban depot and delivers 681 shops located in the city and in the periphery early in the morning. The loading of the vehicles starts from 3 a.m. and is assumed to take 45 minutes. The operations face often tight time windows as shops prefer to receive goods before first customers arrive. The shops are therefore described in terms of time windows and demand (expressed both in volume and weight). The O-D matrices between each shop are also given in terms of time and distance. They were generated based on the location of the shops with the Network analyst of ArcGIS. Finally, we assumed a delivery time of 3 minutes at each shop. Out of these 681 shops, we selected randomly a set of 25, 10, and 5 shops in order to keep reasonable computation times in our analyses.

The generation of instances considered also different set of vehicles. They are based on 10 different vehicles that represent the different vehicle types used in urban freight transport. We consider the segments of the quadricycles (type A), small vans (type B), large vans (type C), and trucks (type D). For each segment, different technologies are represented. The segment of quadricycles considers the diesel Aixam Mega Multitruck (A-d) and the electric Goupil G3 (A-ev). The small van segment uses the electric, petrol, and diesel versions of the Renault Kangoo Express (B-ev, B-p, and B-d). The large van segment is represented by the diesel Mercedes Sprinter (C-d) and the electric Smith Edison (C-ev). Finally, the truck segment uses a diesel and hybrid versions of the Fuso Canter 7.5 tonnes (D-d, D-h). The electric Smith Newton is also considered in that segment (D-ev). The generation of instances considered therefore 7 different sets of vehicles: type A (2 vehicles), type B (3 vehicles), type C (2 vehicles), type D (3 vehicles), BEV only (4 vehicles), Diesel only (4 vehicles), and all vehicles (10 vehicles).

By combining the different sets of vehicles with the different sets of shops, we get a sample of 21 instances to test our algorithm. They are available at the following url: http://mamca.be/plebeau/FSMVRPTW-EV.

## 4. Results

The FSMVRPTW-EV was applied on the 21 instances described above. We used for that a desktop computer with a processor Intel Core i7-2640M and an installed memory of 8 GB. The results of the instances give the total cost and the total distance of the routes. They give also the vehicles that were identified by the FSMVRPTW-EV to achieve the deliveries at a minimal cost. Finally, the computing times to solve the instances are also measured. Results are summarised in Table 2 considering a constant temperature of 15 degrees Celsius.

### 4.1. Algorithm Performance

In terms of computation time, results show that the performance of the algorithm is sensitive to the number of vehicles considered and the number of shops to be delivered. They both increase the number of route combinations that need to be explored in the tree: as the number of shops increases, more vehicles are needed to achieve the distribution and more vehicle combinations are possible.

To a lower extent, complexity of the problems increases also with the capacity of the vehicles considered: the search of the optimal route requires more computation resources to order shops in the route.

### 4.2. Vehicle Classes

When analysing the vehicles classes, Table 2 shows first that the fleet of vehicles cannot be limited to quadricycles as no solutions are found for that class of vehicles. The model does not consider indeed split deliveries: a customer receives one and only one delivery. As a result, some customers have a freight demand that cannot be transported by quadricycles. According to our FSMVRPTW-EV, the potential of quadricycles lies therefore as a complementary vehicle in the mix fleet.

The same conclusion could be drawn for the segment of small vans. When fleet is limited to that class of vehicles, more routes need to be operated because of the limited payload or volume capacity. As a result, distances and costs are more important than in the other vehicle segments. The difference with the quadricycles is that using quadricycles only is not feasible on the instances considered. Using small vans only is feasible but not optimal on the instances considered. Small vans are therefore interesting as a complementary vehicle in the mix fleet (i.e., to deliver specific shops with shorter time windows). The instance with twenty-five shops illustrates this as a small van is selected among all vehicles in the optimal solution: the truck is delivering a maximum of shops and a small van has a capacity large enough to deliver the customers that could not be delivered by the truck. This organisation is reflected on the same instance when we consider only electric vehicles: in that case, a quadricycles is used to deliver the last shops.

The segment of trucks registers most of the time the delivery route with the total minimum distance driven. Their higher capacity requires indeed less vehicles to serve all the customers. However, they are not always selected in the optimal solutions as they have higher costs. In the instances with five shops, the demand is low enough to deliver all customers with a smaller vehicle and a large van. When considering a fleet with only trucks, we can see that they operate the same routing plan than the van. Since the important capacity of the truck is not required, the optimal solution selects the large van. On the other hand, in the instances with ten shops, the truck is selected in the optimal solution. Since demand has increased, the payload of the large van is too limited now to deliver all the customers in one route. Instead, two large vans are used to satisfy the demand while only one truck is required to deliver all the goods. As time windows are large enough to deliver all the shops in one route, the truck is identified to be the best vehicle to use.

### 4.3. Vehicle Technology

As Table 2 shows, the optimal technology varies across instances. In the small instance with five shops, we can see that diesel is preferred. When routing requires a large van to deliver the shops, diesel will be more likely selected over the electric vehicles. Indeed, Lebeau et al. [36] showed that electric vehicles could less compete with diesels in the segment of large vans. Still, the delivery is feasible with a large electric van. The routing plan requires about 25 kWh while the energy capacity of the vehicle is 40 kWh. But it was not selected in the optimal solution as delivering with the large electric van would have entailed distribution with an added cost of 12.5% compared to the optimal solution.

In the larger instance with ten shops, diesel is not selected anymore as the optimal technology. We saw that trucks are more suited for the deliveries given the higher demand. However, in that segment, the hybrid truck is always preferred over the diesel truck by the FSMVRPTW-EV since fixed costs and running costs are lower. Indeed, lower maintenance costs and higher savings from deductibility on taxable profits contribute to lowering fixed costs of the hybrid truck compared to the diesel truck. As a result, hybrid truck is selected most of the time to be the vehicle operating the deliveries at a minimal cost. Let us note that the delivery is also feasible with the electric truck as the energy required to operate that route is estimated to be around 56 kWh while battery capacity is 80 kWh. Hence, limited battery is not a constraint on the operations of the electric truck. But delivering with an electric truck has higher costs and is therefore not selected as the optimal solution. Still, the added cost of delivering with an electric vehicle appears to be lower than in the van segment. The cost of delivering with an electric truck is around 10% higher than delivering with the hybrid truck.

In the largest instance with twenty-five shops, we can see that battery electric vehicles are now part of the optimal solution. Shops are delivered by a hybrid truck and an electric small van. Since the electric small van needs to cover a lower distance than in other vehicle segments to be competitive with diesel van, it is most often preferred over the diesel. For the comparison, delivering the twenty-five shops with a diesel only fleet would require an added cost of 7.5%. On the other hand, delivering the twenty shops with a fleet made of only BEV would require an added cost of less than 2%.

## 5. Conclusions

This paper presents a fleet size and mix vehicle routing problem with time windows for electric vehicles (FSMVRPTW-EV). The main contribution of the authors is considering the variability of range of electric vehicles. Based on real observations, an energy consumption model was developed and integrated in the FSMVRPTW-EV. It is then applied on 21 instances generated from a case in urban freight transport.

The results show that a fleet with different technologies reduces costs of distribution. Indeed, in the segments of quadricycles and small vans, electric vehicles are often the most competitive technology. However, in the segment of large vans, diesel remains the most interesting solution from a financial point of view as electric vehicles would need to cover a very important distance to be cost competitive. Finally, hybrid vehicle is mostly chosen in the segment of trucks as its running costs and fixed costs are lower than the diesel truck. It benefits indeed of higher savings from deductibility on taxable profits and lower maintenance costs. As a result, technology depends especially on the vehicles class required by distribution. When considering the small instance of five shops, one van offers a sufficient capacity to deliver all the shops. Hence, diesel is preferred. When considering the medium instance with ten shops, hybrid vehicle is preferred since one truck is required to deliver the shops with a minimum of vehicles. Finally, in the case of the large instance with twenty-five shops, hybrid and electric vehicles are selected in the optimal set of vehicles as one truck and one small van offer the sufficient capacity to deliver all the shops.

Given the paradox of the electric vehicles, we have seen in our results that electric vehicles are mostly affected by the lower distance bound. Operating a fleet with only electric vehicles fleet implies often higher costs. However, they are barely affected by the upper distance bound since a feasible solution has always been found for instances with only electric vehicles. According to our results, the limited range of electric vehicles is therefore compatible with urban distribution.

Hence, this paper demonstrates the feasibility and the economic relevance of introducing electric vehicles in urban distribution. The vehicle routing problem allows identifying the vehicles that are most adapted to meet customers' requirements. Still, further research should be conducted on the efficiency of the algorithm in order to solve larger problems and meet the needs of daily practice. Also, the energy model could be refined by considering observations on a larger set of electric vehicles.

## Figures and Tables

**Table 1:** Overview of the literature review.

	Fleet size and mix	Time windows	Range constraints	Charging	Energy consumption model
[15]	×	×	✓	×	×
[17]	×	×	✓	×	×
[19]	✓	×	✓	± (location is not considered)	×
[22]	×	✓	✓	✓	×
[20]	×	×	✓	✓ (charging does not depend on the state of charge)	×
[24]	✓	✓	✓	± (location is not considered)	×
[11]	×	✓	✓	✓	×
[25]	✓	✓	✓	✓	×

Source: own setup.

**Table 2:** Results of the FSMVRPTW-EV.

Number of shops	Vehicle set	Computing time (sec)	Total cost (€)	Total distance (km)	Vehicles selected
5	Quadricycles (A-ev, A-d)	—	—	—	—
	Small vans (B-ev, B-d, and B-p)	76	133.24	104.67	B-ev1, B-ev1′
	Large van (C-ev, C-d)	32	103.51	77.88	C-d1
	Truck (D-ev, D-d, and D-h)	48	112.77	77.88	D-h1
	Diesel only (A-d, B-d, C-d, and D-d)	56	103.51	77.88	C-d1
	Electric only (A-ev, B-ev, C-ev, and D-ev)	54	116.45	77.88	C-ev1
	All vehicles (A-ev, A-d, B-ev, B-d, B-p, C-ev, C-d, D-ev, D-d, and D-h)	204	103.51	77.88	C-d1

10	Quadricycles (A-ev, A-d)	—	—	—	—
	Small vans (B-ev, B-d, and B-p)	291	224.2	164.68	B-ev1, B-ev2, and B-ev1′
	Large van (C-ev, C-d)	195	210.17	150.28	C-d1, C-d2
	Truck (D-ev, D-d, and D-h)	483	159.04	123.66	D-h1
	Diesel only (A-d, B-d, C-d, and D-d)	515	168.43	123.66	D-d1
	Electric only (A-ev, B-ev, C-ev, and D-ev)	476	174.47	123.66	D-ev1
	All vehicles (A-ev, A-d, B-ev, B-d, B-p, C-ev, C-d, D-ev, D-d, and D-h)	1630	159.04	123.66	D-h1

25	Quadricycles (A-ev, A-d)	—	—	—	—
	Small vans (B-ev, B-d, and B-p)	3964	389.28	251.31	B-ev1, B-ev2, B-ev3, B-ev4, and B-ev1′
	Large van (C-ev, C-d)	2160	333.97	209.12	C-d1, C-d2, and C-d3
	Truck (D-ev, D-d, and D-h)	5476	309.67	217.28	D-h1, D-h2
	Diesel only (A-d, B-d, C-d, and D-d)	13066	307.22	226.36	D-d1, B-d1
	Electric only (A-ev, B-ev, C-ev, and D-ev)	11235	291.59	204.63	D-ev1, A-ev1
	All vehicles (A-ev, A-d, B-ev, B-d, B-p, C-ev, C-d, D-ev, D-d, and D-h)	140380	285.99	217.28	D-h1, B-ev1

Source: own setup.
